# Nanomaterials-incorporated hydrogels for 3D bioprinting technology

**DOI:** 10.1186/s40580-023-00402-5

**Published:** 2023-11-15

**Authors:** Jungbin Yoon, Hohyeon Han, Jinah Jang

**Affiliations:** 1 Department of Mechanical Engineering, Pohang University of Science and Technology (POSTECH), Pohang, South Korea; 2 School of Interdisciplinary Bioscience and Bioengineering, Pohang University of Science and Technology (POSTECH), Pohang, South Korea; 3 Department of Convergence IT Engineering, Pohang University of Science and Technology (POSTECH), Pohang, South Korea; 4 Institute of Convergence Science, Yonsei University, Seoul, South Korea

**Keywords:** Nanomaterials, Natural ECM hydrogel, 3D bioprinting, Engineered tissue

## Abstract

In the field of tissue engineering and regenerative medicine, various hydrogels derived from the extracellular matrix have been utilized for creating engineered tissues and implantable scaffolds. While these hydrogels hold immense promise in the healthcare landscape, conventional bioinks based on ECM hydrogels face several challenges, particularly in terms of lacking the necessary mechanical properties required for 3D bioprinting process. To address these limitations, researchers are actively exploring novel nanomaterial-reinforced ECM hydrogels for both mechanical and functional aspects. In this review, we focused on discussing recent advancements in the fabrication of engineered tissues and monitoring systems using nanobioinks and nanomaterials via 3D bioprinting technology. We highlighted the synergistic benefits of combining numerous nanomaterials into ECM hydrogels and imposing geometrical effects by 3D bioprinting technology. Furthermore, we also elaborated on critical issues remaining at the moment, such as the inhomogeneous dispersion of nanomaterials and consequent technical and practical issues, in the fabrication of complex 3D structures with nanobioinks and nanomaterials. Finally, we elaborated on plausible outlooks for facilitating the use of nanomaterials in biofabrication and advancing the function of engineered tissues.

## Introduction

Tissue engineering (TE) and regenerative medicine (RM) focus on the creation of functional tissues and organs by combining cells, biomaterials, and bioactive factors [1]. The primary purpose of TE and engineered tissue is to develop biological substitutes that can restore, maintain, or improve the function of damaged or diseased tissue or organs in the human body [2]. Recently, 3D bioprinting has emerged as a cutting-edge biofabrication technology for the fabrication of 3D structures for use in advanced biomedical applications [3]. Furthermore, it allows researchers to design and fabricate structures with a high level of control over the organization of cells, biomaterials, and supportive networks, which allows for the creation of in vitro tissues that closely mimic the structure and function of native tissue [4]. For these reasons, 3D bioprinting technology has tremendous potential, but it still faces various challenges, such as the need for suitable hydrogels, the vascularization of large constructs (i.e., reliable nutrient and oxygen delivery within large-scaled 3D in vitro tissue), and the long-term functionality of bioprinted tissues. Despite this, its advantages mean that it is a promising tool for advanced TE, regenerative medicine, and personalized healthcare.

Extracellular matrix (ECM)-based hydrogels could be employed as a building block for the construction of three-dimensional (3D) tissue structures using 3D bioprinting technology [5]. ECM-based hydrogels are primarily designed to mimic the ECM, a complex network of proteins and other molecules that provides structural support and biochemical cues for cells in the native environment [6]. Therefore, the selection of biomaterials for use in ECM-based hydrogels is crucial for the successful fabrication of engineered tissue. Biomaterials widely used for this purpose include natural polymers such as collagen, gelatin, chitosan, fibrin, alginate, silk, and hyaluronic acid (HA) [7]. Recently, tissue-specific decellularized ECM (dECM) has been widely used for the development of printable hydrogel [7]. The incorporation of natural ECM components into hydrogel s provides several benefits. For example, it facilitates cell attachment, migration, and proliferation, because cells recognize and interact with ECM components in a manner similar to their natural environment [8]. In addition, the natural ECM and dECM-based hydrogels can provide biochemical cues that guide cell behaviors, such as promoting cell attachment, proliferation, and specific cell differentiation pathways, leading to enhanced tissue regeneration, while also contributing to the overall mechanical integrity and stability of the engineered tissue [9].

Although conventional ECM-based bioinks have exhibited significant potential for use in TE and RM applications, they have a number of limitations that researchers are actively addressing. For example, it is often difficult to precisely tune the mechanical properties of printed constructs using conventional ECM-based bioinks [10]. They have risks at clogging the printing nozzle or exhibit low fidelity in reproducing complex tissue architectures [11]. Therefore, various research strategies have focused on developing novel bioink formulations, exploring advanced printing techniques, and integrating bioactive cues to enhance the functionality of conventional ECM-based bioinks for various TE applications. Reinforcing ECM-based hydrogels with nanomaterials to produce nanobioink could be a promising approach to enhance their mechanical properties and expand potential TE applications [12–15]. For example, nanoparticles such as graphene, carbon nanotubes, and clay minerals can enhance the stiffness and strength of bioinks and provide specific bioactive properties, such as drug-delivery capabilities or antibacterial effects, that introduce therapeutic functionalities to the printed tissue [16, 17]. In addition, nanobioink with magnetic nanoparticles can be used to manipulate and align printed constructs with the application of an external magnetic field [18]. Similarly, polymer nanofibers, such as collagen or cellulose, can provide a scaffold for tissue regeneration due to their improved structural support and mechanical integrity and promote cell adhesion to the surface area of the construct [19].

**Fig. 1:**
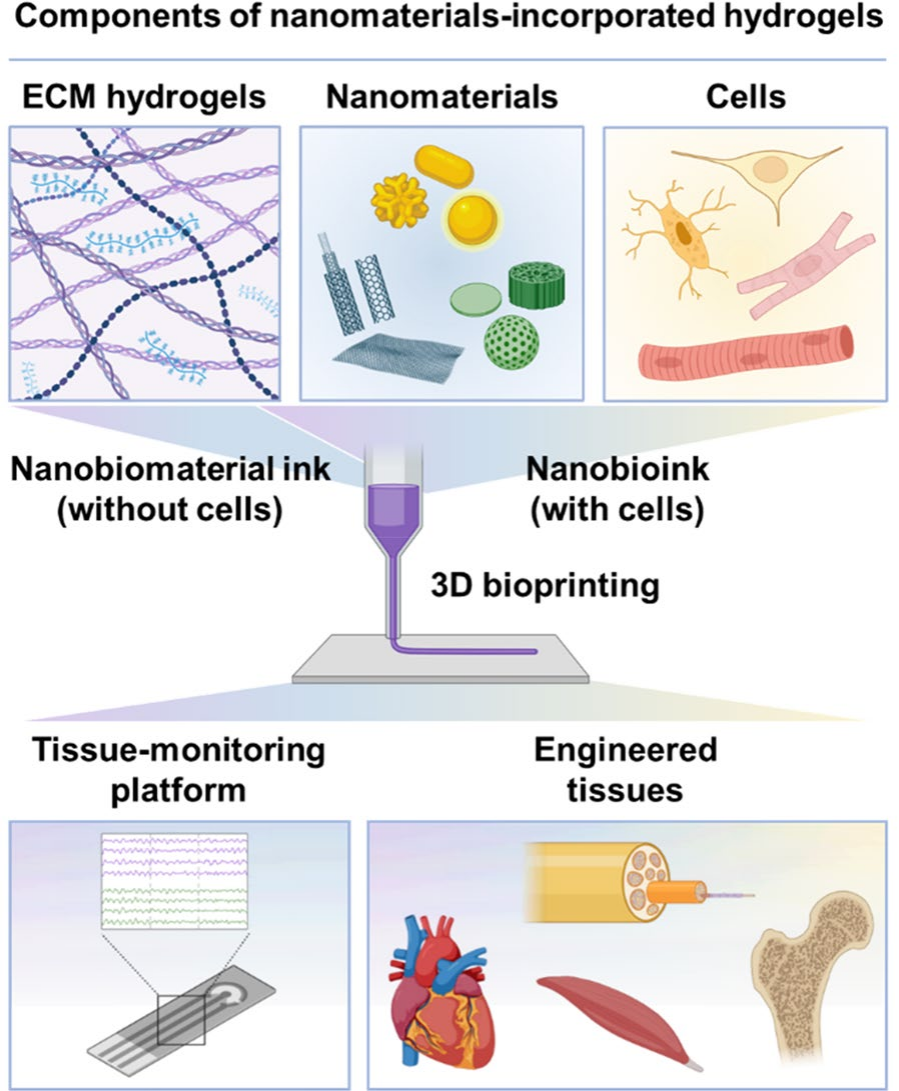
The definition of nanobiomaterial ink and nanobioink depending on the cell component and the scope of this review is illustrated. Created with BioRender.com

In this paper, we comprehensively review nanomaterial-incorporated ECM hydrogels (i.e., nanobioink) that have been introduced to 3D bioprinting technology for successful TE and RM (Fig. 1). Initially, we address the limitations of the natural ECM hydrogels, such as low printability and low structural fidelity, hence reduced functional performance of engineered tissue, then introduce various nanomaterials that have been used to enhance the mechanical properties (during printing) and structural fidelity (after printing) of natural ECM hydrogels. Finally, we summarize past and current TE research using nanobiomaterial and nanobioink with 3D printing technology and discuss future directions for the development of more innovative printable bioink and the enhancement of the functional performance to create more native tissue-like geometries and microenvironments within bioprinted 3D structures.

## Natural ECM hydrogels for 3D printing technology

### Collagen

Collagen is the most abundant protein group in the human body, accounting for approximately 25–30% of the total vertebrate protein [20]. Collagen represents a group of at least 28 protein isoforms, with type I collagen a major component of skin, bones, and connective tissues and the principal fibrillar constituent of the ECM in the mammalian body [21]. Collagen proteins have the ability to initiate or control various cellular functions and processes, including cellular differentiation, motility, communication, and programmed cell death [22]. There is substantial evidence supporting the clinical use of collagen across a wide range of applications in TE [20, 21, 23–25]; hard tissues [26], such as bone [27–33], cartilage [34–36], and meniscus [37], and soft tissues such as vascular tissue [38–40], cardiac tissue [41, 42], adipose tissue [43], neural tissue [44–46], skin [47–51], and corneal tissue [52, 53], in addition to its use in drug delivery [54]. Although collagen generally has excellent biocompatibility, biodegradability, and cytocompatibility, the poor mechanical strength and structural stability of collagen scaffolds impose limitations on its use for specific tissues. To enhance the mechanical properties of collagen, intermolecular cross-linking using physical or chemical techniques, such as modifying with methacrylate groups to produce photo cross-linkable collagen methacryloyl (ColMA or MeCol) has been introduced.

### Gelatin

Gelatin is a natural biopolymer based on the hydrolysis of type I collagen. The use of different collagen sources and preparation techniques results in gelatin products with various physical properties and chemical heterogeneity [55]. Due to its structural similarity to collagen, gelatin is characterized by favorable biocompatibility, poor mechanical properties, and rapid biodegradation [56]. In addition, gelatin has low cytotoxicity and immunogenicity compared to collagen and is generally recognized as safe by the Food and Drug Administration (FDA) [57]. Gelatin is also bio-adhesive, which is attributed to the presence of cell-binding motifs, specifically arginine-glycine-aspartic acid (RGD) peptides. These functional RGD sequences enhance cell adhesion, differentiation, and proliferation [56, 58, 59]. Although gelatin stands out for its numerous advantages, its low thermal stability at physiological temperatures remains a significant drawback [60].

Consequently, gelatin methacryloyl (GelMA) hydrogels were designed to have chemical stability and physical integrity at physiological temperatures [58, 61–63]. Currently, GelMA is one of the most versatile hydrogel platforms due to its unique combination of bio-functionality and mechanical tunability, and it can be easily customized by adjusting the polymer composition, polymer concentration, and cross-linking density [64]. The robust application of GelMA in TE has been the subject of multiple recent reviews [58, 65–68]. Nevertheless, the use of GelMA as a hydrogel is still restricted by its low viscosity, limited range of conditions suitable for biofabrication, and cell damage that occurs during UV cross-linking [69–72]. In this context, an alternative visible-light photoinitiation system using ruthenium and sodium persulfate has been developed, demonstrating improved viability of encapsulated cells and shape fidelity [73–77].

### Chitosan

Chitosan, a polysaccharide derived from partially deacetylated chitin found in crustacean shells, shares structural similarities with glycosaminoglycan, a component of ECM [78]. Chitosan is a particularly attractive biomaterial due to its high availability, biodegradability, biocompatibility, non-toxicity, hydrophilicity, antimicrobial and antifungal properties, ability to promote wound healing, and, most importantly, its versatility [79, 80]. The free amine groups present in the backbone chain of chitosan can be modified either chemically or physically to promote elasticity, flexibility, and a lower inflammatory response [79, 81, 82]. In addition, various chitosan derivatives capable of photopolymerization, such as chitosan methacrylate (ChiMA or MAC), have been developed recently [83–87]. These unique properties make chitosan a promising biomaterial for numerous biomedical and TE applications; however, to date, the application of chitosan and its derivatives in TE has mostly concentrated on a few tissue types such as bone [88–100], skin [101–110], and nerves [111–118], possibly because chitosan has been underrated due to its insolubility in aqueous solutions [119].

### Alginate

Alginate is a natural anionic polysaccharide derived from brown algae [120]. Alginate hydrogels are widely used in various biomedical and TE applications due to their favorable biocompatibility, biodegradability, and gelation [121]. These hydrogels are formed by cross-linking alginate chains to create a 3D network structure. The cross-linking process for alginate is typically achieved by adding divalent cations, such as calcium ions from calcium chloride, that act as bridges between the alginate molecules [122]. The cross-linking process also creates a gelatinous structure that can hold significant water while maintaining its integrity [123]. For this reason, cross-linked alginate in bioink guarantees the stability and stiffness of 3D-printed constructs [124].

Alginate hydrogels have advantages when used in biomedical applications. They can mimic the ECM found in natural tissues and provide a supportive environment for cell growth and proliferation [125]. The porosity and mechanical properties of these hydrogels can be tuned to resemble specific tissue types, making them suitable for tissue regeneration and wound-healing applications [126]. These alginate hydrogels also can encapsulate and deliver bioactive molecules, such as drugs or growth factors, in a controlled manner [127]. The alginate matrix can protect the encapsulated molecules from degradation and release them gradually, allowing for sustained therapeutic effects [128]. Moreover, alginate hydrogels are often used as bioinks in 3D bioprinting due to their shear-thinning behavior, which enables the precise deposition of cells and biomaterials during the printing process [129]. Overall, alginate hydrogels have a wide range of applications in TE, drug delivery, wound healing, and 3D bioprinting, making them a versatile and promising material in biomedical research. Nevertheless, the alginate hydrogels are still not strong enough to maintain the structure and shape long-term; alginate in 3D printed architecture tends to collapse due to its low viscosity; therefore, an efficient solution is required to enhance the printability of alginate hydrogel.

### Hyaluronic acid

HA is a naturally occurring polysaccharide found in the ECM of various tissues in the human body, including the skin, umbilical cord, and vitreous humor [130]. It plays an important role in tissue hydration, lubrication, and cell signaling in the body [131]. HA consists of repeating disaccharide units of D-glucuronic acid and N-acetylglucosamine, a structure that offers excellent water-binding properties [132]. To create HA hydrogels, HA molecules are typically chemically modified to introduce cross-linking sites, which allow the formation of a stable gel structure. This cross-linking can be achieved through various methods, including physical (e.g., temperature or pH-induced gelation) or chemical approaches (e.g., using Schiff’s base, enzyme-mediated photopolymerization, Michael-type addition, disulfide formation, and click chemistry reactions) [133].

The resulting HA hydrogel provides a 3D scaffold with a structure that mimics the ECM of human tissues. This structure provides the cellular functions of adhesion, proliferation, and migration, meaning that HA-derived hydrogels are attractive for use in TE applications, such as wound healing, cartilage repair, and drug delivery [134]. Moreover, incorporating bioactive molecules, such as growth factors or drugs, into HA-derived hydrogels can further enhance their therapeutic potential [135]. Specifically, the porous structure of an HA hydrogel allows the controlled release of molecules by adjusting the in vivo degradation of HA. Therefore, HA hydrogel properties enable localized drug delivery and promote tissue regeneration in therapeutic applications [136]. HA is particularly well-suited for 3D bioprinting applications because of its strong influence on various biological functions (e.g., cytokine stimulation [137], angiogenesis [138], cell attachment [139], and cell proliferation [140]). Collectively, HA-derived hydrogels are versatile biomaterials with excellent biocompatibility and tunable properties for 3D bioprinting, demonstrating great promise for use in various biomedical TE, cosmetic, and dermatological applications.

Though HA-derived hydrogel offers exceptional biological properties, its printability and post-printing stability are relatively low due to its high swelling [13]. Thus, HA is mechanically unstable and often undergoes rapid hydrolytic degradation due to oxidation [141]. In addition., the hydrophilic nature of HA limits cell adhesion [142]. Thus, chemically modified cross-linkable HA scaffolds are required for fine-tuned TE after controlling key properties of the hydrogel, such as its printability, post-printing stability, cell damage, cross-linking density, and porosity [141].

### Silk fibroin

Silk fibroin (SF) is a protein found in silk fibers [143]. SF is derived from the cocoons of silkworms and has been widely studied and utilized as a biomaterial in TE due to its close resemblance to ECM components such as collagen and because of its associated advantages, including high biocompatibility, tunable biodegradation, minimal immunogenicity, and mechanical resilience [144]. SF hydrogels exhibit several desirable properties for biomedical applications, including excellent mechanical strength and the maintenance of their structural integrity under physiological conditions [145]. These hydrogels are also highly biocompatible, meaning they are tolerated by living tissue and do not induce significant inflammation or immune responses [146]. In addition, its suitability for aqueous processing and ability to be produced in different formats have encouraged the use of SF in hydrogel for 3D bioprinting applications [147]. In particular, SF hydrogels have been demonstrated to be very useful in developing self-standing structures for use in cartilage, bone, and skin TE [148]. These promising results have led to further research on the use of SF to develop multifunctional bioinks that incorporate additives for biological and physicochemical enhancements when seeking to regenerate complex 3D tissue architectures [149].

The porous architecture of SF hydrogels on a 3D-printed scaffold also allows for the diffusion of nutrients, oxygen, and waste products, making it suitable for supporting cell growth, cell proliferation, and tissue regeneration. SF hydrogels can also be loaded with bioactive molecules, such as growth factors or drugs, and used in controlled drug release/delivery systems and wound-healing therapeutics. Despite the excellence of SF hydrogel, the simple cross-linking method of SF hydrogel yielded poor printing [149]. Therefore, to explore the improved potential of SF in developing multifunctional hydrogels and to enhance its biological and physicochemical relevance in regenerating complex 3D tissue architectures, incorporating additives to SF hydrogel has to be further considered.

### Decellularized extracellular matrix

Tissue-derived dECM has gained significant attention over the past few decades as a biomaterial for TE and various biomedical applications [150]. The decellularization process removes cellular components from tissues or organs, resulting in an acellular scaffold, using chemical (i.e., dissolving the cell membrane, lipids, and proteins via detergence), physical (i.e., removing cells by mechanical force), and enzymatic (i.e., degrading nucleic acids, proteins, and lipids with enzymes) approaches [151]. dECM is made up of a complex arrangement of structural proteins such as collagen, laminin, elastin, and fibronectin, which provides the mechanical rigidity and structural stability necessary for cellular adhesion, growth, migration, and proliferation [9]. Other components in dECM, such as glycoproteins, proteoglycans, and bound growth factors, mediate its morphological organization and physiological functions [9]. Additional components in dECMs, such as glycoproteins, proteoglycans, and bound growth factors, mediate morphological organization and physiological function [152]. At the same time, glycosaminoglycans (GAGs) create an extremely hydrophilic environment, which is essential for withstanding high compressive force [153].

dECM hydrogels have been extensively studied for use in TE and RM, including in the production of bioinks for the manufacturing of 3D structures [154]. In particular, dECM-based hollow tubes and bifurcating structures resembling anatomical features such as blood vessels, kidney tubules, and airways have been 3D printed [155, 156]. The variety of tissues from which dECM can be extracted determines the versatility and functionality of the bioprinted structures, with which intrinsic cellular morphologies and functions can be reconstituted [157]. In addition, 3D dECM-based scaffolds enhance cell growth and differentiation, facilitating tissue repair and regeneration. In addition, by providing a biocompatible and bioactive environment, dECM hydrogels can guide cell behavior and promote the formation of functional 3D bioprinted tissues [158]. However, still, there are the weak aspects of dECM hydrogel, which are low printability and shape fidelity; therefore, the incorporation of nanomaterials became a promising exploration to enhance the mechanical properties, printability, and structural integrities of dECM hydrogel.

## Utilization of nanobioinks in 3D tissue engineering

### Collagen and chitosan-based nanobioinks and engineered tissues

In TE and RM, restoring volumetric muscle loss is a major priority. Kim et al. developed a collagen-based nanobioink where gold nanowires (GNWs) were incorporated to produce in vitro muscle tissue (Table 1) [159]. Adjusting various printing parameters enabled the alignment of the GNWs in the printing direction, and applying an external electrical field to the printed tissue induced a uniaxial orientation in the GNWs. The aligned GNWs provided topological cues to the cells and accelerated the alignment of myoblasts while mimicking the electrical properties of muscle tissue. In addition, type I collagen is commonly used in cardiac TE because it is a major component of cardiac ECM; however, its electrical conductivity and mechanical properties need to be tailored for successful integration with the host tissue and to withstand the stretching-contracting stress from the beating heart following implantation. Izadifar et al. employed methacrylated collagen, ColMA (or MeCol), and carboxyl functionalized CNTs to produce a bioink, with the addition of CNTs resulting in a highly interconnected nanofibrous structure and higher conductivity, particularly within a frequency range relevant to physiological conditions [160]. They also encapsulated human coronary artery endothelial cells in the nanobiomaterial composite ink, creating a lumen-like vascular structure in the printed patch, highlighting the ability of the hydrogel microenvironment to induce an appropriate cellular response.

**Table 1:** Summary of nanobioinks based on collagen, chitosan, and their methacrylated forms used to fabricate in vitro tissues

Base ECM	Nanocomponent	Function of the nanocomponent	Printed shape	Application	Refs.
Collagen	Gold nanowires (GNWs)	Aligned topological cues (supplemented with external electric field) to the myoblasts	Porous lattice structure	Muscle tissue regeneration	[159]
ColMA (or MeCol)	Carboxyl-functionalized carbon nanotubes (CNTs)	Mechanical reinforcer Electrical conductivity enhancer	Rhombic porous structure	Cardiac patch for myocardial infarction repair	[160]
Limitations of collagen-based systems	Widely used in muscle fabrication; however, there is a need for improvement in electrical conductivity and mechanical properties				
Chitosan	Nanohydroxyapatite (nHAp)	Osteogenic cue	Disc shape	Bone tissue	[92]
Methacrylated chitosan (MAC or ChiMA)	Nanosilicate (Laponite®)	Mechanical reinforcer (compressive modulus ~ 15 MPa) Biomineralization cue	* Porous structure		[181]
Limitations of chitosan-based systems	Application is hindered due to limited solubility under a physiological (neutral) environment				

*: Scaffolds were printed first, then the cells were seeded

Many studies have focused on the fabrication of hard tissues, such as bone, by adopting a strategy of printing scaffolds in advance and subsequently seeding them with cells [161–164]. This is because, for example, the incorporation of nanomaterials such as nanohydroxyapatite (nHAp) into a hydrogel can enhance the viscosity of the ink and thus increase printability, but this also poses a challenge in terms of achieving a stable dispersion, potentially leading to nozzle clogging; This can impact not only the printing quality and mechanical properties of the printed constructs but also the cells encapsulated in the hydrogel [165, 166]. As nHAp is one of the most commonly used nanomaterials in bone and cartilage tissue engineering [167, 168], various efforts have been made to address the clogging problems. Demirtaş et al. addressed this issue by selecting a suitable hydrogel system for the homogeneous dispersion of nHAp [92]. They prepared a nanocomposite hydrogel of nanostructured bone-like nHAp and an alginate or chitosan hydrogel, respectively, and compared the suitability of the pure hydrogels and the composite hydrogels as nanobioinks for bone TE. As a result, chitosan was the base hydrogel of choice. The abundant amino groups in the backbone of chitosan can also be protonated, producing a positive charge when dissolved in aqueous solutions. This strengthens the interaction between the nanomaterial and the chitosan polymer network compared with the neutral hydrogel, facilitating the homogeneous dispersion of nanomaterials with a negatively charged surface (e.g., CNTs), within the chitosan hydrogel [169]. Furthermore, the surface modification or grafting of nanoparticles with chitosan polymer chains is recognized as a practical approach to enhancing nanoparticle dispersion [170, 171]. nHAp generally exhibits a negative surface charge in its unmodified state due to the presence of phosphate groups, meaning that it exhibits good dispersion in a chitosan hydrogel solution [172, 173].

ChiMA, a methacrylated form of chitosan, has been utilized for a broad range of biomedical applications [66, 67], including drug delivery [176, 177], dental restoration [178, 179], and wound healing [177], due to its bioadhesiveness and antimicrobial activity, with more recent applications in bone TE [180, 181]. For example, Cebe et al. introduced nanosilicate particles to ChiMA to augment the compressive strength of 3D-printed bone tissue scaffolds, demonstrating a maximum compressive strength of approximately 15 MPa, which approaches the compressive strength of cancellous bone [181]. Using this reinforced hydrogel, the scaffolds were printed as mesh-like structures, and osteoblast precursor cells were subsequently seeded onto the scaffold. Greater formation of biominerals in the bone tissue was observed after culturing for 21 d.

### Gelatin/GelMA-based nanobioinks and engineered tissues

Gelatin offers many excellent properties for use as a hydrogel, such as good biocompatibility, low antigenicity, and support for cell adhesion and proliferation; however, its potential for 3D printing is limited by its low mechanical strength [188, 189]. Nanocellulosic materials such as cellulose nanocrystals (CNCs) and nanofibers (CNFs) can be employed as either a reinforcing agent or a blending modifier to enhance the mechanical strength of hydrogel bioinks [10, 11]. Jiang et al. utilized dialdehyde cellulose (DAC) nanocrystals, an oxidized form of CNC derived from a plant, as a natural cross-linker (Table 2) [184]. DAC interacts with gelatin (or chitosan or collagen) through Schiff’s base reactions without generating toxic residues and enhances the mechanical strength of gelatin by creating a dense network structure [190–192]. Blending DAC into collagen increases the breaking strength of nanobiomaterial ink about 41-fold compared with pure gelatin. This DAC-reinforced gelatin hydrogel generates porous 3D scaffolds with good fidelity. However, this method may not be suitable for high-throughput fabrication due to the extended cross-linking time (> 24 h) needed to attain the desired viscosity before printing.

**Table 2:** Summary of gelatin/GelMA-based nanobioinks used to fabricate various in vitro tissue types

Base ECM	Nanocomponent	Function of the nanocomponent	Printed shape	Application		Refs.
Gelatin or GelMA	Dialdehyde cellulose (DAC) nanocrystal	Natural cross-linker to enhance the mechanical properties of gelatin hydrogel	* Porous scaffold in different shapes (circle, regular hexagon, square)	Tissue repair (not specific)		[184]
	TEMPO-oxidized cellulose nanofibrils (CNFs)	Viscosity regulator and facilitator for the cross-linking of GelMA	Porous lattice and simple disc structure	Wound-healing scaffold		[185]
	nHAp	Osteoconductive factor Mechanical reinforcer	* Tri-layered hierarchical scaffold	Bone and cartilage	Osteochondral tissue	[161]
			* Porous lattice scaffold		Bone tissue	[162]
	Nanosilicate	Printability enhancer Osteoinductive cues Potential vehicle for drug retention and delivery	Porous lattice structure		Angiogenic bone tissue	[69]
		Osteoinductive cues	Pyramidal constructs containing a perfusable vasculature inside		Vascularized bone tissue	[182]
	Graphene nanoplatelets	Mechanical reinforcer Neuronal differentiation cues	Porous lattice structure (stereolithography-based printing)	Electro-active tissues	Neural tissue	[186]
	GNRs	Electrically conductive bridges connecting electroactive cardiomyocytes	30-layered constructs with inner grids (direct printing) and spiral constructs (embedded printing)		Cardiac tissue	[183]
	Gold nanoparticles (AuNPs)	Printability enhancer Electrically conductive bridges connecting electroactive myoblast	Dot shape	Skeletal muscle tissue construct		[187]
Limitations of gelatin-based systems	Despite its extensive utilization across various biomedical fields, its relatively weak mechanical properties limit its application in specific applications (i.e. load-bearing tissues)					

*: Scaffolds were printed first, then the cells were seeded

**Fig. 2.**
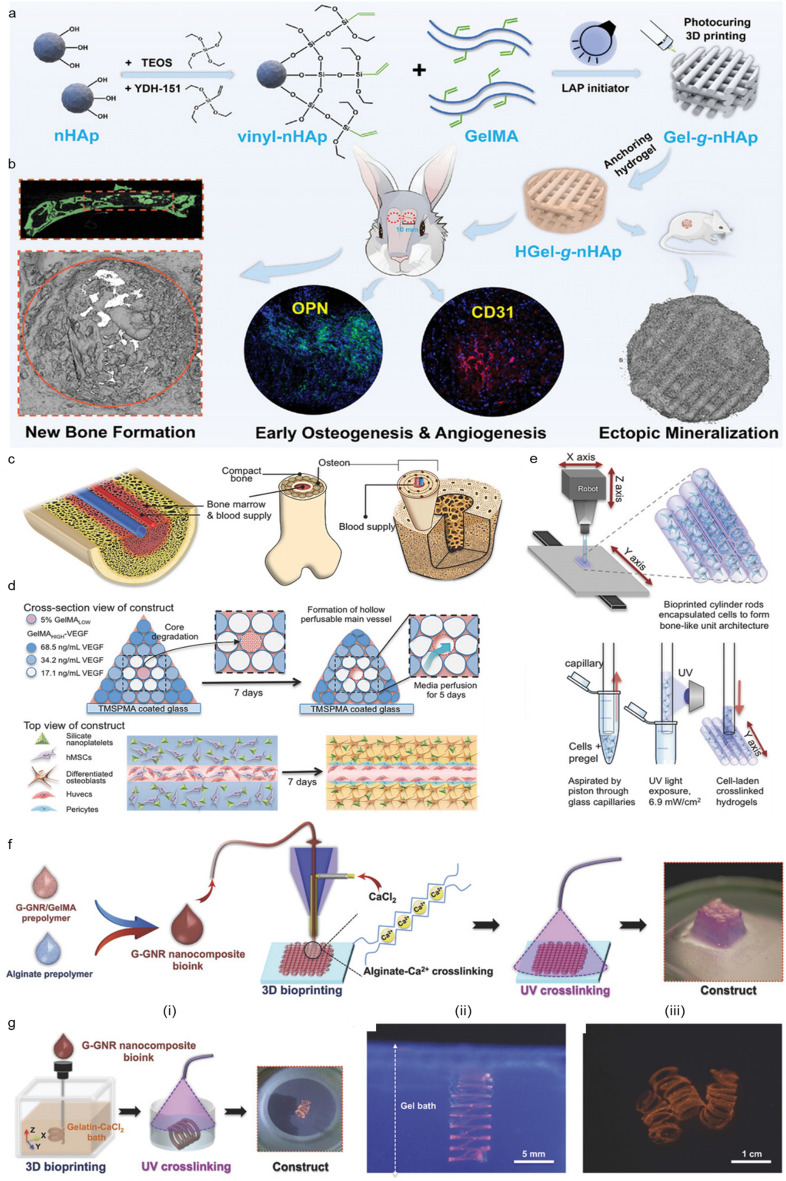
3D bioprinted in vitro tissue constructs using GelMA-based nanobioinks.** a**, **b** Development process for a tailored nHAp-based GelMA bioink for efficient osseointegration and bone regeneration: **a** schematic diagram of the surface modification of nHAp and coupling with GelMA and **b** bone regeneration via the nanostructured 3D printed hydrogel scaffold. Reprinted with permission from [162]. **c–e** Fabrication of 3D vascularized bone mimetic architecture: **c** schematic illustration of complex native bone structure, **d** bioprinting strategy for the fabrication of bone tissue with perfusable vasculature, and **e** 3D printing process for the fabrication of pyramidal constructs from cell-laden cylindrical rods. Reprinted with permission from [182]. **f,g** Bioprinting strategies used to fabricate cardiac tissue constructs with gold nanorod (GNR)-incorporated GelMA: **f** lattice construct fabricated via the layer-by-layer stacking of coaxially printed GNR-GelMA nanocomposite bioink, **g (i)** embedded bioprinting using a support batch to fabricate a spiral construct, with printed spiral constructs **(ii)** in the support batch and **(iii)** in a culture medium. Reprinted with permission from [183]

GelMA, the methacrylated form of gelatin, offers rapid and robust biofabrication with its photo cross-linking capabilities leading to gelation within a few seconds to a few minutes. Incorporating nanomaterials into GelMA makes it possible to fine-tune its mechanical and viscoelastic properties, impart electrical and magnetic functionalities, and stimuli-responsiveness on the system [66, 67, 193]. For example, Xu et al. printed a fibroblast-laden scaffold for wound-healing therapy using low concentrations of GelMA (≤ 1% (w/v)) containing nanocellulose [185]. In their study, in addition to the intrinsic cross-linking capability of GelMA, the strong physical interaction between negatively charged CNF and GelMA and the in situ cross-linking of CNF via Ca^2+^ facilitated the successful 3D printing of low-concentration hydrogels into freestanding 3D scaffolds with excellent structural stability. Liu et al. also designed a tri-layered osteochondral scaffold that mimics the intricate and hierarchical structure of native osteochondral tissue via the 3D printing of GelMA/nHAp at different concentrations with a top cartilage layer of 15% (w/v) GelMA, an intermediate layer of 20% (w/v) GelMA and 3% (w/v) nHAp (20/3% GelMA/nHAp), and a bottom subchondral bone layer of 30/3% GelMA/nHAp [161]. Similarly, Tong et al. modified the surface of nHAp to add vinyl groups and chemically integrated it with GelMA via photoinitiation to create a gelatin-grafted nHAp scaffold [162]. As a result, the gelatin-grafted nHAp replicated the mineralized collagen fiber nanostructure of natural bone and improved the dispersion of nHAp and its limited interfacial interaction with the matrix hydrogel compared with simple blending (Fig. 2a, b). Nevertheless, printing nHAp particles with cells remains a challenge; in both cases above, acellular GelMA/nHAp biomaterial ink was printed into scaffolds first, and cells were seeded later because the cells would be damaged by the high shear stress arising from the pressure required to extrude the nanobiomaterial ink (120 kPa-300 kPa) [194, 195].

Synthetic silicate nanoplatelets have demonstrated promise for use in bone TE because they degrade into nontoxic products such as Na^+^, Mg^2+^, Si (OH)_4_^+^, and Li^+^ in aqueous solutions, thus promoting specific cellular reactions to facilitate the induction of osteogenesis [196–198]. For example, Laponite® is a disc-shaped synthetic nanosilicate with a thickness of 1 nm and a diameter of 25 nm, that has a positive charge at the rim and a negatively charged surface. In addition, aided by visible-light photoinitiation, Cidonio et al. produced a GelMA bionink with a high shape fidelity and drug retention capacity [69]. The addition of Laponite® (0.5 and 1.0 wt%) considerably enhanced the printability of the nanobioink, promoting filament formation and stacking. The dual-charged surface of Laponite® was expected to contribute to the greater drug/protein localization capacity, but drug-release tests with lysozyme and bovine serum albumin (BSA) as analogs for bone morphogenetic protein-2 and vascular endothelial growth factor (VEGF) showed that the presence of Laponite® did not lead to significant differences in the release profile of BSA. Byambaa et al. focused on the incorporation of a large-scale, perfusable vasculature within a bone construct to prevent tissue necrosis that can occur in volumetric tissue [182]. Two GelMA hydrogel bioink formulations were optimized to support vasculogenesis and osteogenesis and printed into individual cylinders. A pyramidal vascularized bone construct was printed by stacking these cylinders with the vasculogenic hydrogel used to print the central vessel and the silicate nanoplatelets-loaded osteogenic nanobioink was used to print the surrounding bone (Fig. 2c-e).

Regeneration of nerve tissue is one of the most common goals in TE. Replacement of diseased or injured nerves or bridging the nerve gap caused by tissue loss relies on autografts. However, this gold-standard therapy suffers from several problems, such as donor tissue shortage, morbidity at secondary surgery sites, and unsatisfactory regeneration. To overcome these issues, the development of tissue-engineered nerve grafts has been studied. Various ECM materials, including HA, alginate, chitosan, collagen, SF, gelatin, and GelMA, have been combined with nanomaterials for nerve TE [199–208]. For example, in one study conducted by Zhu et al., neural stem cells (NSCs) and a graphene nanoplatelet were encapsulated in GelMA to produce a composite hydrogel precursor solution, which was then used to fabricate a 3D scaffold using UV-assisted stereolithography-based 3D printing [186]. In the 3D hydrogel scaffold, the graphene nanoplatelets induced the proliferation and differentiation of NSCs into neuronal cells. Although the mechanisms underlying the graphene induction of cellular neurogenesis are still poorly understood [209], recent studies have suggested that graphene can regulate the bioelectric properties of cellular membranes during the crucial phases in the development of NSCs [210]. In addition, key factors such as the formation of focal adhesions on graphene and environmental stimulation from graphene may also play governing roles in the modulation of neurogenesis [211].

Diverse applications have been proposed for GelMA and its nanomaterial composites, with one of the most recent applications being in the context of cardiac and skeletal muscle. In these electroactive tissue types, the electrical conductivity of the matrix surrounding the cells is critical, because these electrical cues are vital for cellular growth and development [212]. Hence, generating a conductive hydrogel bioink is essential to emulate the native ECM environment and reduce the electrical insulation that could hinder intercellular electrical propagation. Furthermore, electrically conductive nanomaterials based on carbon [213–216], graphene oxide (GO) [217–220], and gold [221–223] have demonstrated their potential to promote the maturation and organization of cardiomyocytes in in vitro cardiac tissue constructs. The nanomaterials utilized in the fabrication of skeletal muscle tissue share similarities in terms of purpose and type with those used for cardiac muscle. In particular, various gold nanostructures, such as nanospheres, nanorods, and nanowires, have been recognized as highly promising electrically conductive nanomaterials for biomedical research due to their biocompatibility, ease of fabrication and modification, and diverse aspect ratios [224, 225]. Numerous studies have utilized gold nanostructures in cardiac TE [183, 222, 226]. For instance, Zhu et al. developed a GNR-blended GelMA-based hydrogel for the 3D printing of cardiac tissue constructs [183]. The incorporation of GNRs improved the electrical conductivity of the hydrogel by bridging the electrically resistant hydrogel pore walls, leading to enhanced cell-to-cell coupling and the coordinated contraction of the cardiac constructs (Fig. 2f-h). Boularaoui et al. also incorporated AuNPs into GelMA to enhance the printability and conductivity of hydrogel bioink [187]. The myoblast-laden composite hydrogel was printed into skeletal tissue, and enhanced myosin heavy chain expression and myotube elongation were observed in the presence of AuNPs.

### Alginate-based nanobioinks and engineered tissues

Alginate hydrogels are still not sufficiently strong to maintain their structure and shape in the long term due to their low viscosity, leading to their collapse in 3D-printed architecture. The viscosity of alginate hydrogels can be enhanced by raising the concentration and molecular weight of the alginate in the bioink; however, this method does not allow for sufficient shape fidelity during 3D bioprinting [127]. Furthermore, alginate hydrogels maintain fewer bioligands and exhibit limited bioactivity, such as reduced cell adhesion [128]. Therefore, alginate-derived hydrogels need to be modified by the addition of other materials to improve the mechanical properties of the resulting bioink and increase the structural fidelity of the 3D bioprinted construct (Table 3). In this respect, nanofibrillated cellulose (CNF) is an excellent rheological modifier with shear-thinning and dispersant properties that can promote improved ECM printability.

**Table 3:** Summary of alginate-based nanobioinks used to fabricate various in vitro tissue types

Base ECM	Nanocomponent	Function of the nanocomponent	Printed shape	Application	Refs.
Alginate	CNF	Excellent rheological modifier with shear-thinning and dispersant properties to improve ECM printability	Grid shaped scaffold	Adipose tissue	[227]
	Polylactic acid (PLA) nanofiber	ECM hydrogel stiffness enhancer Enhanced cell adhesion sites	Strand and meniscus-shaped construct		[228]
	CNF	Printing resolution enhancer Shape fidelity enhancer Encapsulated cell viability enhancer	Multiple geometries (grid, human ear-like shape, sheep meniscus-like shape)	Cartilage tissue	[229]
			Chondrocyte-encapsulated 3D gel disc		[230]
			Single layered lattice structure		[231]
			Simple disc structure		[232]
	Multiwalled carbon nanotubes (MWNTs)	Bioprintability enhancer	Perfusable, co-axial printed vessel-like structure	Vessel	[233]
	Nanosilicate	Shape fidelity enhancer Encapsulated cell viability enhancer Favorable drug delivery ability	Porous scaffold in different shapes (Tube- and cubic- like constructs)	Bone tissue	[234]
Limitations of alginate-based systems	Alginate-based systems in bioprinting exhibit limitations such as structural weakness, especially in 3D-printed structures, and limited bioactivity due to fewer bioligands and reduced cell adhesion				

Saljo et al. mixed alginate hydrogel with CNF to print 3D adipose tissue [227]. The combination of alginate and CNF produced the highest storage modulus, thus exhibiting the most gel-like properties with improved mechanical characteristics. The enhanced printability of the composite alginate/NFC hydrogel and the CaCl_2_-based cross-linking process allowed the fabrication of a solid grid architecture after printing [227]. This structure also immobilized human adipose progenitor cells and promoted differentiation into adipocytes. In addition, a 3D printed 10-mm diameter half-sphere (height: 3 mm) construct was grafted; after 30 d in vivo, novel blood vessels were observed on the graft surface, with signs of angiogenesis in the graft and vascularization in the center of the tissue [227]. Narayanan et al. mixed alginate with PLA nanofibers and human adipose-derived stem cells (hASCs) to characterize an alginate–nanofiber bioink for cell growth and mature tissue formation [228]. Combining PLA nanofibers and alginate in a printing process offered unique advantages in terms of mimicking the native ECM and providing a suitable environment for cell growth and tissue formation. The PLA nanofibers reinforced the alginate hydrogel and enhanced its structural integrity, thus allowing for the fabrication of intricate geometries and promoting higher levels of cell proliferation within the bioprinted construct, with a peak observed on day 7 and most cells remaining viable on day 16. The metabolic activity of encapsulated hASCs on day 7 was 28.5% higher in the PLA-nanofiber-reinforced alginate bioink compared with the bioink without nanofibers. These results indicate that bioinks with PLA nanofibers offer a more beneficial environment for hASCs, thus aiding in their proliferation.

**Fig. 3.**
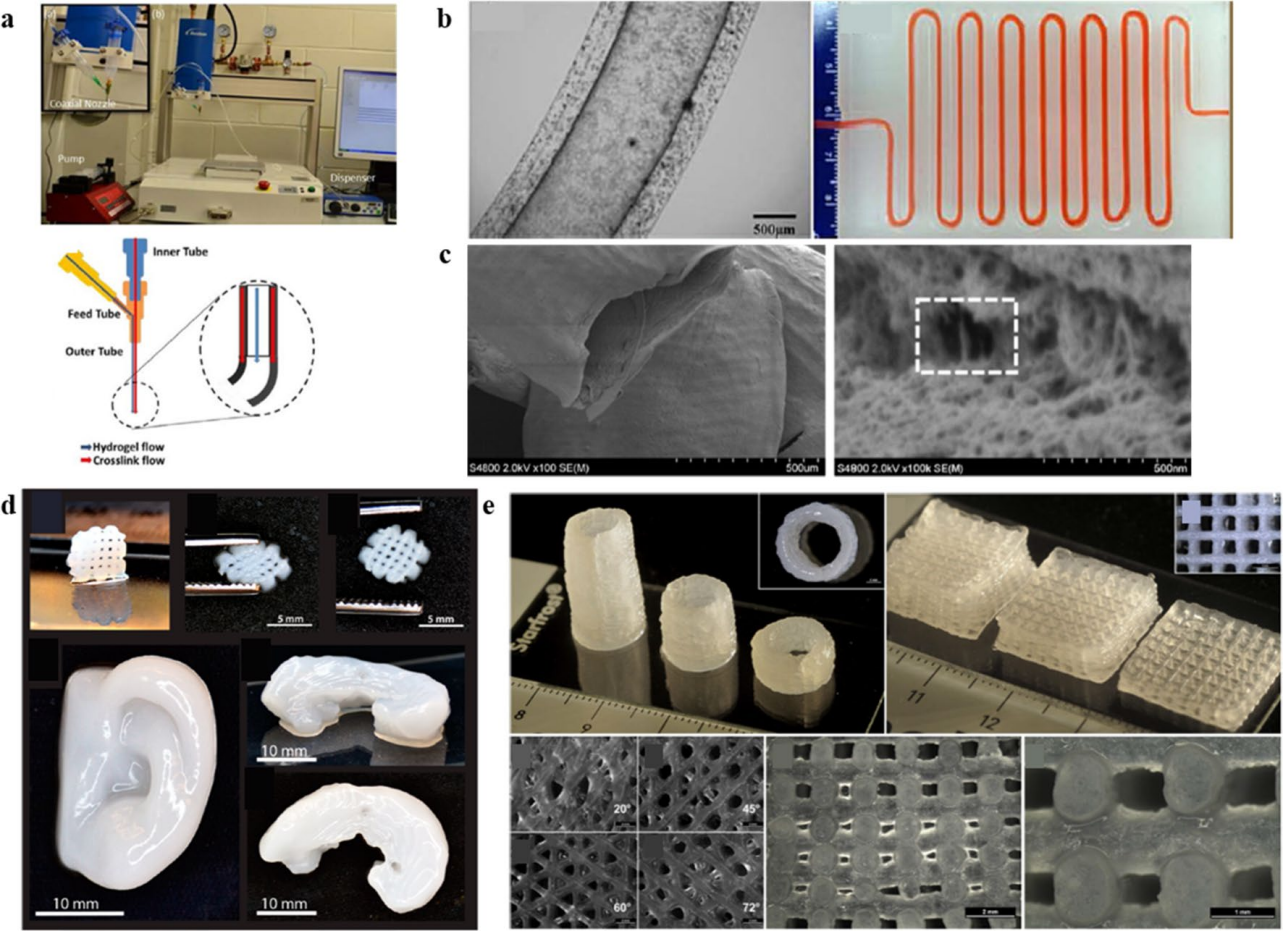
3D bioprinted tissue constructs using alginate-derived nanobioinks. **a**–**c** Construction of perfusable vessel conduits with MWCNT reinforced alginate nanobioink. **a** Experimental setup for the fabrication of vascular conduits based on a coaxial nozzle system. b Printed perfusable vascular conduits. **c** SEM images of a vascular conduit printed using a 4% alginate ECM hydrogel reinforced with 1% MWCNTs. The image shows the structural integrity of the tubular shape. Reprinted with permission from [233]. **d** Various 3D printed structures using alginate–CNF bioink. Reprinted with permission from [229]. **e** Development of various printed structures using bioinks combining 3% (w/v) Laponite®, 3% (w/v) alginate, and 3% (w/v) methylcellulose. Reprinted with permission from [234]

Dolati et al. modified the properties of alginate by CNTs or MWNTs in the fabrication of perfusable vascular conduits (Fig. 3a,b) [233]. CNTs are known to reinforce biocompatible alginate polymers to increase their strength and resistance against the deformation of the composite vasculature [3, 6, 7]. In particular, MWNTs have a high mechanical strength, stiffness, and aspect ratio, while also being lightweight, flexible, and low-density [10]. Furthermore, because vascular conduits derived from low-concentration alginate hydrogels collapse easily, CNT reinforcement has the potential to enhance the mechanical, structural, and perfusion characteristics of these conduits while offering biologically favorable properties [16]. CNT reinforcement has been reported to increase the ultimate tensile stress by 11% and the modulus of elasticity by 94%, while decreasing the maximum strain by 18%. Similarly, MWNTs have a high affinity for alginate-based matrices, significantly enhancing their mechanical properties and bioprintability [22, 23]. The formation of this matrix in the inner surface of a 3D vascular conduit also enables pulsatile media to flow through the lumen of a co-axial cell printed structure **(**Fig. 3c), allowing encapsulated human coronary artery smooth muscle cells to proliferate in the pulsatile flow, as seen in natural blood vessels [233]. However, though acceptable cell viability was observed in the CNT-reinforced conduits in short-term cultures, only a limited number of cells survived and functioned properly in long-term cultures [233].

The CNFs, such as bacterial nanocellulose fibrils, offer hydrophilicity, facile chemical modification, and a high surface area [235]. In addition, bacterial nanocellulose fibrils are microscopically similar to collagen fibrils, with a similar width of about 100 nm, facilitating their use as an additive to alginate hydrogel in 3D printed scaffolds for the engineering of soft tissue such as cartilage [236]. Markstedt et al. added CNFs and human chondrocytes to alginate-based bioink to print multiple geometries (grid, human-ear, and sheep-meniscus shapes) at a high printing resolution (Fig. 3d) [229]. Their nanocellulose − alginate bioink was non-cytotoxic, with human chondrocytes encapsulated on the designed 3D printed structures exhibiting a cell viability of 73% and 86% after 1 d and 7 d of 3D cultures, respectively [229]. Muller et al. also created a nanocellulose − alginate hydrogel and mixed it with human chondrocytes to print gel discs [230]. Overall, their nanocellulose − alginate bioink exhibited improved printing properties and lower shear stress (with the use of 413 μm in a conical needle), while enhanced cell proliferation, cell spreading, and collagen II synthesis were also observed on the encapsulated human chondrocytes in the printed 3D gel discs [230]. Nguyen et al. bioprinted induced pluripotent stem cells (iPSCs) in an NFC/alginate composite hydrogel for cartilage regeneration [231]. For the 3D bioprinted 60% NFC/40% alginate construct, pluripotency was initially maintained for five weeks, with enhanced collagen type II expression and the attenuated expression of tumorigenic gene observed [231]. The directed chondrogenic differentiation of the iPSCs offers a potential molecular pathway for the optimization of articular cartilage tissue generation.

Based on the results above, the 3D bioprinting of iPSCs with CNF/alginate hydrogels offers a promising therapeutic strategy for the repair of damaged cartilage in human joints. Continuously, combining the shear thinning properties of CNF with the fast cross-linking ability of alginate becomes a promising hydrogel for 3D bioprinting with living cells for the growth of cartilage tissue. Following these advances, Möller et al. printed a 5 × 5 × 1 mm scaffold made up of human nasal chondrocytes, human bone marrow-derived mesenchymal stem cells, CNF, and an alginate hydrogel [232]. This 3D-printed scaffold was transplanted into a subcutaneous pocket of a mouse. Consequently, 60 d after implantation, ECM synthesis and human collagen II deposition were enhanced, indicating successful in vivo chondrogenesis [232]. Therefore, a bioink composed of CNF and alginate is a suitable biomaterial for 3D bioprinting with living cells to produce mature 3D in vitro cartilage tissue.

Ghadiri et al. showed that a combination of Laponite® and alginate increased the viscosity of an injectable hydrogel and improved the Young’s modulus of the resulting cross-linked scaffolds [237]. Furthermore, Laponite®–alginate hydrogels have been shown to sustain the release of cationic drugs such as doxorubicin with improved release kinetics [238]. Jin et al. also reported that a high concentration of Laponite®–alginate hydrogel had enhanced printability, with improved biocompatibility with encapsulated cells and greater shape fidelity for printed construct also observed [239]. Ahlfeld et al. also printed human mesenchymal stem cell-laden scaffolds made up of combinations of 3% (w/v) Laponite®, 3% (w/v) alginate, and 3% (w/v) methylcellulose (also known as 3–3-3 scaffold) **(**Fig. 3e**)** [234]. Following extrusion, approximately 70–75% of the printed immortalized human mesenchymal stem cells survived, and cell viability was maintained over 21 d within the constructs [234]. Moreover, the release of growth factors exhibited a more sustained profile with the inclusion of Laponite® in the 3–3-3 scaffolds when compared with an alginate–methylcellulose blend [234]. In conclusion, the combination of Laponite®, alginate, and methylcellulose illustrates the great potential of biomaterials as robust, scalable, and reliable bioinks for successful 3D printing in skeletal TE.

### Hyaluronic acid-based nanobioinks and engineered tissues

Even though HA is an appealing biomaterial for TE, it remains limited by its mechanical properties and degradation rates, thus restricting its clinical applications [133]. Nevertheless, HA can be chemically modified or physically cross-linked to improve its mechanical properties and control its degradability [240]. Additionally, reinforcing HA hydrogels with nanoparticles, such as natural CNCs, can produce a composite material with improved mechanical properties [241]. CNCs can be extracted from a wide range of highly available cellulose sources and offer a high elastic modulus (110–220 GPa) and tensile strength (7.5–7.7 GPa), a low density, a high aspect ratio, a high surface area, and improved biocompatibility, thus enhancing their suitability for use in RM and TE applications [242]. Specific surface modifications such as oxidation, esterification, etherification, silylation, and polymer grafting can also be used to effectively incorporate CNCs into an HA-based hydrogel matrix, which can act as a filler and cross-linker [243]. A summary of nanomaterial-reinforced HA hydrogels is provided in Table 4.

**Table 4:** Summary of HA-based nanobioinks used to fabricate various in vitro tissue types

Base ECM	Nanocomponent	Function of the nanocomponent	Printed shape	Application	Refs.
HA	CNFs	Printing resolution enhancer Shape fidelity enhancer Adipogenesis promoter	Simple disc structure	Adipose tissue	[244]
	nHAp	Osteogenic cues	Porous grid structure	Bone tissue	[245]
	Carboxymethylcellulose (CMC)	Shear-thinning enhancer Self-healing cues	Various printed structures (lattice, cubic, and tube shapes)	Soft tissue (not specified)	[13]
	2D Ti_3_C_2_ MXene nanosheets	Electroconductive inducer	Porous scaffold	Neural tissue	[246]
	Lignin nanoparticles	Electroconductive inducer Promoted neuronal differentiation cues	Porous scaffold	Spinal cord injury repair	[247]
Limitations of HA-based systems	HA-based systems encounter issues with mechanical strength, controlled degradation influenced by enzymatic activity and inflammation, and biocompatibility, while their structural simplicity poses challenges for tissue integration, cellular interaction, biological response control, and scalable, reproducible fabrication, particularly in 3D bioprinting				

Because nanocellulose can be conjugated with proteins to facilitate cell attachment, Henriksson et al. employed it to analyze in vitro adipocyte activity [244]. The authors prepared Cellink-H bioinks containing nanocellulose and HA with a dry weight ratio of 70:30 or 80:20 [250]. Before 3D bioprinting, the bioinks were gently mixed with a mouse-derived mesenchymal stem cell suspension to achieve a final concentration of 10–30 million (M) cells per 1 ml of Cellink-H bioink. The bioinks were printed in a three-layered gridded structure in each well of a 24-well plate. After one week of 3D construct culturing, cell viability was 95%. Furthermore, after two weeks, the viable cells displayed a more mature phenotype with larger lipid droplets than standard 2D cultures [250]. In particular, unlike the cells in the 2D cultures, the 3D bioprinted cells did not detach with lipid accumulation. In addition, the gene expression of the adipogenic marker genes increased 2.0- and 2.2-fold for cells in the 3D bioprinted constructs compared with 2D cultured cells after two weeks of incubation [250].

Wenz et al. prepared a bioink containing modified methacrylated HA/gelatin and HA modified with nHAps (5% w/v) [245]. In their study, primary hASCs were encapsulated in the HAp-containing methacrylated HA/gelatin hydrogel, and printed constructs were cultured for 28 d, resulting in a higher storage moduli (126 ± 9.6%) compared to the initial value on day 1[254]. The distinct change in the elastic and viscous hydrogel properties were attributed to extensive bone matrix production in the hASC-encapsulated HAp-containing methacrylated HA/gelatin hydrogels. In particular, bone-matrix-related components such as collagen I, fibronectin, alkaline phosphatase, and osteopontin were highly expressed in the printed grid construct after 28 d of culturing [254]. The authors thus successfully developed an osteoinductive bioink that enabled the bioprinting of a 3D composite matrix as a precursor for bone tissue development based on hASCs.

**Fig. 4.**
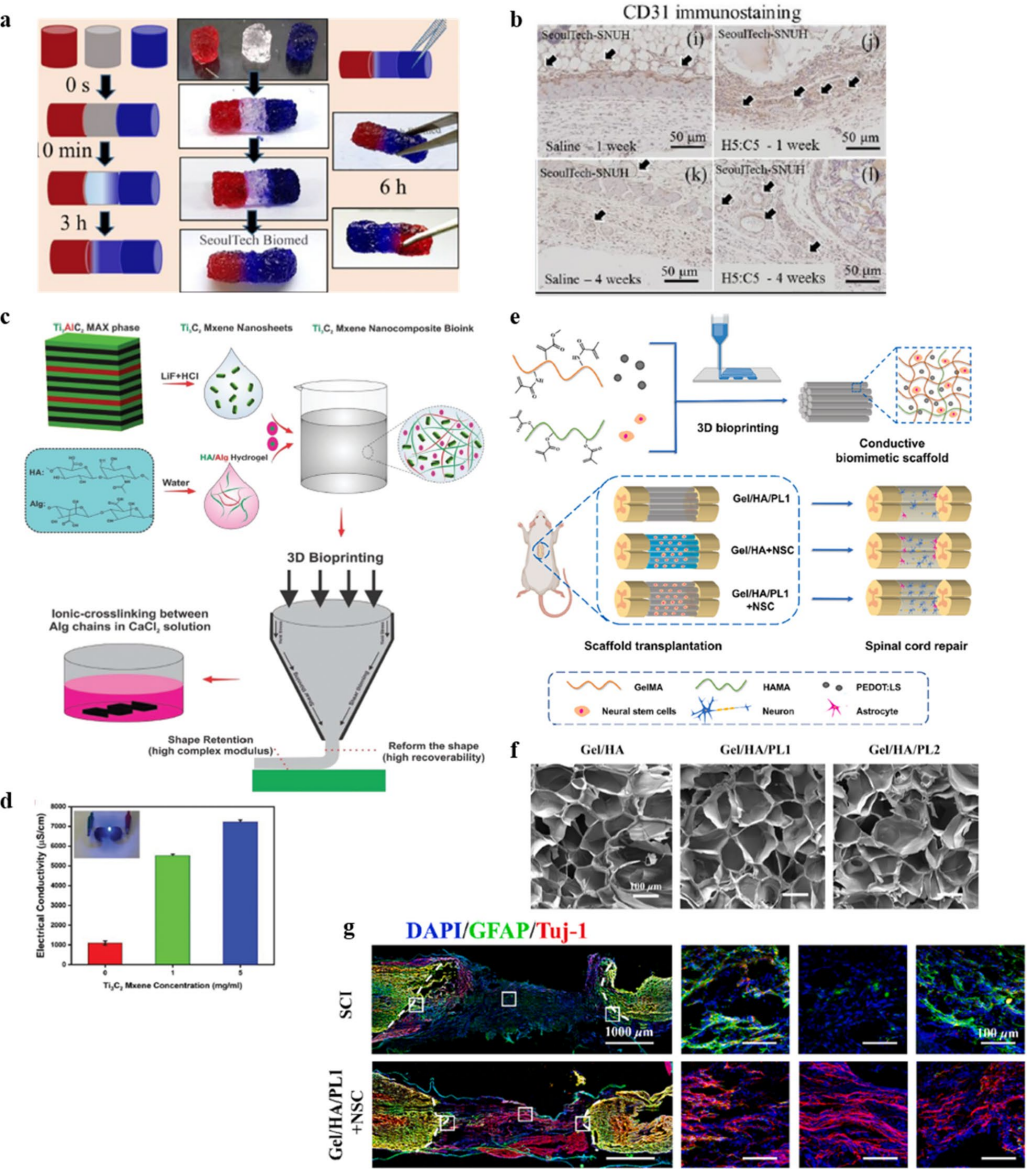
3D bioprinted in vitro tissue constructs using HA-based nanobioinks. **a**,** b** Development of 3D printable HA-CMC nanobioink-derived scaffolds. **a** Investigation of the self-healing capacity of HA-CMC hydrogels over time and their mechanical stability. **b** Angiogenic marker CD31 immunostaining after 1 and 4 weeks at the implanted site of an HA-CMC hydrogel printed scaffold in a mouse model. Reprinted with permission from [13]. **c**, **d** Examination of electroconductive HA-MXene nanobioink** c** Schematic representation of the synthesis of an MXene-based nanocomposite bioink. **d** Characterization of the MXene nanocomposite bioink in terms of electrical conductivity. Reprinted with permission from [246]**. e–g** Establishment of the neural capacity of Gel/HA/PL nanobioink derived spinal cord biomimetic scaffolds. **e** Schematic representation of 3D bioprinted conductive spinal cord biomimetic scaffolds for the promotion of the neuronal differentiation of neural stem cells and the repairing of spinal cord injury. **f** Characterization of the porosity of Gel/HA/PL hydrogels. **g** Characterization of implanted conductive biomimetic scaffolds in terms of neurogenesis. Reprinted with permission from [247]

To overcome previous printing issues (e.g., gel printability, post-printing stability, cell damage, and toxic cross-linker), Gopinathan et al. developed a self cross-linking microporous HA–CMC hydrogel through chemically stable acyl-hydrazone bonding chemistry [13]. As a result, CMC has been commonly used to improve the mechanical properties and strength of HA hydrogels [248]. Furthermore, acyl-hydrazide chemistry prevents rapid degradation due to the formation of stable acyl-hydrazone bonds through the reaction between HA-mono-aldehyde and CMC-hydrazide to enhance hydrolysis. Gopinathan et al. mixed the mono-aldehyde HA (HA-mCHO) and acyl-hydrazide functionalized CMC (CMCCHZ) together to form stable HA-CMC hydrogels via acyl-hydrazone bonds under normal physiological conditions [13]. The acyl-hydrazone grafted between two polysaccharide structures provided much-needed stabilization of the hydrogel, while the mono-aldehydes did not affect the HA backbone. The physiochemical properties, pore size, and printability of the HA-CMC hydrogels were also investigated. Tissue regeneration requires a controlled 3D environment that enables cells to migrate, proliferate and differentiate with adaptable or self-healing cross-links [241]. To examine the self-healing abilities of the HA-CMC microporous hydrogels, Gopinathan et al. placed three hydrogel samples (red, white, and blue) together and observed that the hydrogels remained separate with distinct boundaries; however, the attached gels started healing after 10 min (Fig. 4a) [13]. Under optimized conditions, the HA-CMC hydrogels were used to fabricate self-standing 3D structures in lattice, cube, and tube designs of up to 50 layers without any support materials or post-cross-linking process [13]. Furthermore, quercetin (a model anti-oxidative drug) was slowly released from a 3D-printed hydrogel at a 15% lower rate than the non-printed hydrogel. The 3D-printed constructs (pore size ~ 50 μm) formed denser networks due to the higher stress experienced during extrusion through the needle; thus, they exhibited slower drug release over 96 h [13]. Specifically, in vivo*,* subcutaneous experiments revealed that the HA-CMC hydrogels had excellent angiogenetic effects (enhanced CD31 expression after the subcutaneous injection of the HA-CMC hydrogel) without toxicity (Fig. 4b**)** [13]. These advantages may facilitate the fabrication of more intricate biological structures using 3D bioprinting, mimicking native tissues and organs with a homogeneous distribution of biofactors within multiple layers.

The electrical conductivity of nanomaterial-reinforced ECM hydrogels has proven advantageous for improving the signaling between cells [249]. However, conventional bioinks consisting of polymeric biomaterials suffer from inherently poor electrical conductivity due to their chemical structure [249]. Previous studies have shown that the incorporation of electrically conductive materials (e.g., metal nanoparticles, carbon-based nanomaterials, and conductive polymers) within the hydrogel bioink can enhance the conductivity of the resulting bioink, which enables the propagation of electrical signals between cells [186]. For example, Rastin et al. developed a human embryonic kidney 293 cell (HEK-293)-laden electroconductive bioink composed of 2D Ti_3_C_2_ MXene nanosheets dispersed within an HA/alginate derived hydrogel for extrusion-based 3D bioprinting (Fig. 4c) [246]. Increasing the Ti_3_C_2_ MXene content in the bioink improved the electrical conductivity (Fig. 4d), while the highly thixotropic behavior of the HA/Alg hydrogel toward the 2D Ti_3_C_2_ MXene nanosheets improved the printability of the bioink [252]. Over a week, the cell-laden bioink exhibited relatively low cytotoxicity, and the results of live/dead cell assays revealed high cell viability (> 95%) for the 3D bioprinted structures. These results suggest that MXene nanocomposite bioinks and 3D bioprinting technology, which offer high electrical conductivity, biocompatibility, and degradability, are useful for neural TE [246].

Spinal cord injury (SCI) involves serious damage to the central nervous system and, according to the World Health Organization (WHO), up to 500,000 people worldwide are disabled with SCI each year [250]. Currently, the clinical treatment modalities for SCI include surgery, medication, and rehabilitation but, despite the remarkable progress in treatment techniques, restoring the sensory and motor functions of SCI patients remains a significant challenge [251]. In recent years, the development of neural TE technologies has led to the proposal of new solutions for accelerating the recovery of SCI patients [252]. Recently, Chen et al. developed novel spinal cord biomimetic scaffolds using 3D bioprinting technology with a conductive hydrogel consisting of hyaluronic acid methacrylate (HAMA), GelMA, and poly(3,4-ethylene-dioxythiophene) doped with sulfonated lignin (PEDOT: LS) (Fig. 4e) [247]. LS has previously been used to effectively dope PEDOT and improve the dispersibility and electrical conductivity of PEDOT. PEDOT: LS has also proven to have excellent biocompatibility [253]. The HA/Gel/PLS hydrogel in printed scaffolds exhibited a porous morphology with an average pore size of about 100 μm (Fig. 4f) [247]. This type of porous morphology is critical for inducing the proliferation and differentiation of NSCs [254]. The conductive spinal cord biomimetic 3D scaffolds were subsequently implanted into a rat spinal cord complete transection injury model, with the 3D printed platform strongly promoting the neuronal differentiation of NSCs in vitro with greater neuronal regeneration (increased Neuron-specific class III beta-tubulin [Tuj-1] signals) in the injured section and a large amount of glial scar tissue (increased Glial Fibrillary Acidic Protein [GFAP] signals) forming around the cavity (Fig. 4g) [247].

### Silk-based nanobioinks and engineered tissues

The mechanical and structural properties of SF hydrogels can be tailored by increasing the SF concentration, using cross-linking, and/or adjusting the post-printing processing conditions. However, despite these efforts, cross-linked SF hydrogels still have a printing resolution that is too low (average hydrogel stiffness of 2.5–5.0 kPa a shear strain of 30%) to meet the requirements of native TE [185]. For example, implanted SF hydrogel scaffolds need a stiffness ranging in the kPa- to GPa- range to match native natural tissues such as muscles and tendons [255]. For skeletal muscle, the typical stress and strain are 100 kPa and 20%, respectively [263], while the equilibrium modulus and dynamic stiffness are 0.27 and 4.10 MPa, respectively, for native articular cartilage explants [256]. Thus, the mechanical properties of SF-derived scaffolds need to be reinforced to meet the requirements for the development of functional 3D in vitro tissues. For example, Rodriguez et al. established a novel 3D freeform fabrication strategy and method of SF using a Laponite® and PEG suspension, inducing the *in-situ* physical cross-linking of SF without the need for additives or post-processing [257]. This development allows the *in-situ* physical cross-linking of pure aqueous SF into defined geometries produced through freeform 3D printing.

Nanofibers have a large surface-to-volume ratio, a high surface area, exceptional mechanical properties, a low cost, and outstanding biocompatibility, while their width of about 100 nm is similar to that of collagen fibrils derived from ECM. These superior characteristics of nanofibers are suitable for the fabrication of a 3D environment similar to that of native tissue. Taking advantage of bacterial cellulose nanofibers in SF/gelatin-based bioink, Li et al. printed nanocomposite scaffolds with improved shape fidelity; in particular, the SF/gelatin-BCNF bioink maintained a good shape, allowing soft tissue models to be printed [258].

Mimicking the heterogeneity of phases and anisotropic intricacy observed for native osteochondral interfaces has been a significant challenge for osteochondral TE. However, a previous study has shown that including hierarchically biomimetic strontium-doped nano-apatites as a ceramic additive to silk-based bioink can be used to promote osteoinduction and the osteocyte maturation of encapsulated stem cells [143]. The same study also found that the porous nature of the silk-based network within 3D bioprinted osteochondral constructs enables better diffusion and promotes cellular communication, hence forming wavy boundaries at the interface between the chondral and bone phases [152]. Moses et al. also reported evidence that strontium-based bone bioinks favored the activation of M2 macrophage activation, thus opening possible routes for exploring the immunomodulatory effects of these bioinks.

In addition, to promote in vitro bone regeneration, Patel et al. printed silk fibroin/chitosan/cellulose nanoparticle (SF/CS/CNP) scaffolds using a bio-printer [259]. The resulting 3D-printed biodegradable SF/CS/CNP scaffolds demonstrated improved rheological features and enhanced recovery strength, leading to greater osteogenic differentiation potential. The higher expression of osteogenic proteins was also observed in the SF/CS/CNP-derived scaffolds compared to an SF-only hydrogel, highlighting the superior osteogenic potential of the developed scaffolds [267]. The 3D printed SF/CS/CNP scaffolds further facilitated M2 macrophage differentiation and triggered the secretion of different growth factors and proteins to promote bone regeneration via the macrophage-mediated process [267]. A summary of 3D-printed, SF-based nanocomposites developed for use in TE is provided in Table 5.

**Table 5:** Summary of silk-based nanobioinks used to fabricate various in vitro tissue types

Base ECM	Nanocomponent	Function of the nanocomponent	Printed shape	Application	Refs.
SF	Bacterial CNFs	Printing resolution enhancer Shape fidelity enhancer	Porous scaffold	Soft tissue (not specified)	[258]
	CNPs	Bone regeneration cues Excellent biocompatibility Superior mechanical strength M2 macrophage polarization	Porous scaffold	Bone tissue	[259]
	Sr^2+^ doped nHAp	Cues of osteogenesis, chondrogenesis, and angiogenesis	Bilayered scaffold (lower layer: bone; upper layer: cartilage tissue)		[143]
Limitations of Silk-based systems	Silk-based material is hindered by the insufficient mechanical properties of its printed 3D scaffolds, which do not meet the diverse stiffness requirements of native tissues (e.g., muscles and tendons)				

### Decellularized Extracellular Matrix (dECM) -based nanobioinks and engineered tissues

Despite the close compositional resemblance of dECM hydrogels to native tissues, the solubilization processes employed for their production (i.e., the use of proteolytic enzymes to induce matrix digestion) damage the native structure of their fibrous proteins [260]. This reduces their biomechanical stiffness and increases their biodegradability, limiting their long-term functionality. In particular, the principal thermal cross-linking mechanism in dECM is often insufficient for maintaining its structural stability within 3D constructs due to the weak hydrogen bonding with the digested components in the solubilized dECM matrix [261]. Therefore, dECM hydrogels can be combined with other natural polymers, such as gelatin, chitosan, alginate, HA, and SF, or alternative photo-cross-linking methods can be employed (e.g., methacryloyl modification) to improve their mechanical rigidity [262]. For example, methacryloyl-modified kidney- and bone-specific dECM bioinks have demonstrated greater mechanical stability, with tunable cross-linking possible with the use of moderate ultraviolet or blue-light irradiation [262]. dECM hydrogels can also be modified to incorporate additional bioactive molecules, such as growth factors or drugs, to enhance their therapeutic potential [263]. These modifications can be achieved by functionalizing the dECM hydrogel with specific chemical groups or incorporating nanoparticles or microspheres within the 3D hydrogel structure [264]. Overall, dECM-derived hydrogels offer a promising approach for TE, RM, and drug delivery applications due to their biomimetic nature and ability to support cellular activities in a controlled manner (Table 6**).**

**Table 6:** Summary of dECM and additive nanobioinks used to fabricate various in vitro tissue types

Base ECM	Nanocomponent	Function of the nanocomponent	Printed shape	Application	Refs.
Methacryloyl-modified SIS dECM (SISMA)	Fully exfoliated GO nanosheets	Electrostimulation of 3D bioprinted tissue constructs	Porous lattice structure	Constructing 3D bioprinted tissue (Not specified)	[265]
SIS dECM	Sr^2+^/Fe^3+^ co-doped nHAp	Osteogenic and angiogenic cues	Multilayered lattice scaffold	Bone tissue	[266]
Bone dECM	MWCNTs	Cardiac contractility enhancer	hPSC-CM-laden 3D scaffold	Cardiac tissue	[267]
Skin dECM	Electrospun PLGA nanofibrous sheet + Skin-derived dECM nanofiber scaffold	Wound-healing properties Tissue regeneration cues	Bilayer (upper: epidermis layer; lower: dermis layer) membranous (BLM) nanofiber scaffold	Skin tissue	[268]
Limitations of dECM-based systems	Despite their compositional similarity to native tissues, the solubilization and thermal cross-linking processes in dECM hydrogel production impair the native fibrous protein structures and long-term structural stability, respectively				

**Fig. 5:**
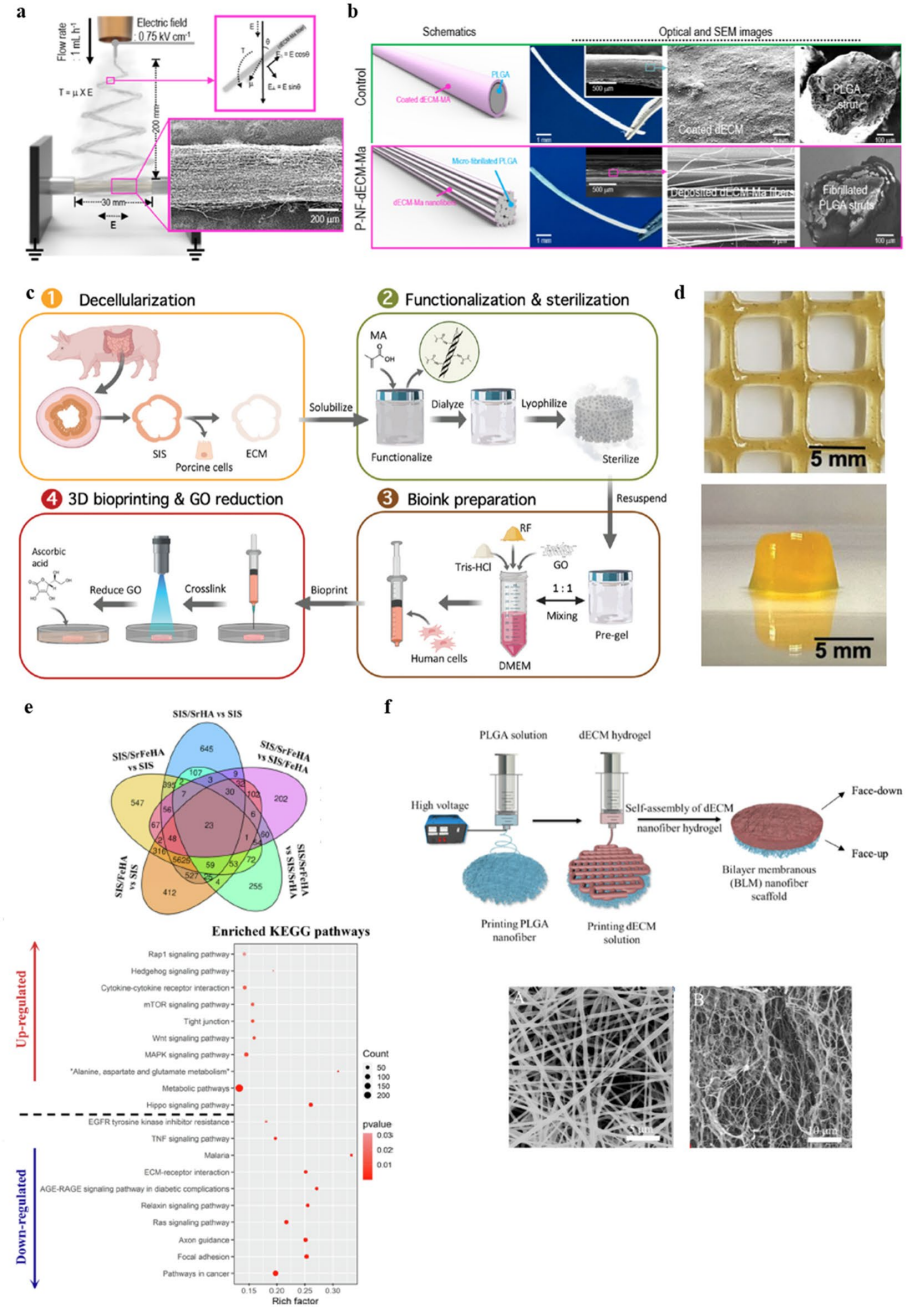
Various tissue engineering using dECM-based nanobioinks via 3D printing technique.** a**, **b** Development of electrospun dECM and 3D printed PLGA combined scaffold. **a** Schematic diagram of electrospinning on fibrillated PLGA struts and fibers aligned using electrostatic torque. **b** Schematics, optical images, and surface and cross-sectional SEM images of control (upper panel) and P-NF-dECM-MA scaffolds (lower panel). Reprinted with permission from [269]. **c**, **d** Procedure for preparation of 3D printable SISMA-GO nanobioink. **c** Preparation of an SISMA-GO composite bioink and its bioprinting schematic. **d** Characterization of the shape fidelity of bioprinted SISMA-GO composite hydrogels. Reprinted with permission from [265]. **e** RNA sequencing analysis of HUVECs cultured on SIS/SrFeHA scaffolds for 7 days, and a Venn diagram of differentially expressed genes between groups (upper panel) and enriched Kyoto Encyclopedia of Genes and Genomes (KEGG) pathways based on differentially expressed genes for SIS/SrFeHA vs. SIS (lower panel). Reprinted with permission from [274]. **f** Manufacturing process for a BLM (PLGA nanofiber layer and skin-dECM nanofiber hydrogel layer) nanofiber scaffold via 3D printing. Images of the surface morphology of the PLGA nanofiber layer (left) and dECM nanofiber hydrogel layer (right) are shown in the lower panel. Reprinted with permission from [268]

Lee et al. chemically modified skeletal muscle-specific dECM using a methacrylate reaction (dECM-MA) to improve its structural stability before electrospinning [269]. The electrospun dECM-MA nanofibers were combined with 3D printed, microscale fibrillated poly(lactide-co-glycolide) (PLGA) constructs (P-NF-dECM-MA scaffolds) to promote skeletal muscle cell orientation and maturation (Fig. 5a,b) [277]. Electrospinning and 3D printing-mediated micropatterning techniques have been widely used to fabricate aligned structures similar to the anisotropic arrangement of stretched myofibers found in skeletal muscle and provide topographic cues for morphogenesis [270, 271]. In Lee et al.’s study, the multiscale P-NF-dECM-MA scaffolds significantly promoted cellular orientation, with the aspect ratio of the F-actin alignment considerably higher than in the control scaffold due to the contact guidance of the uniaxially aligned multiscale structure [269]. In addition, compared to the control scaffold with nonaligned MHC, the uniaxially arranged myosin heavy chain (MHC) on the P-NF-dECM-Ma scaffold demonstrated enhanced myogenic differentiation and promoted myotube formation [277]. Therefore, combining biochemical cues from dECM-MA and topographical cues from a 3D printed, multiscaled scaffold exhibits great promise for the development of functional 3d bioprinted skeletal muscle tissue that could potentially be used for RM applications.

GO is a flexible nanomaterial that has attracted significant attention due to its high hydrophilicity [279] and ability to enhance protein absorption from culture media and improve cell–hydrogel interactions by offering more cell adhesion sites [272]. Rueda-Gensini et al. used exfoliated GO nanosheets with methacryloyl-modified, small intestine submucosa-derived dECM (SISMA) to allow for the electrostimulation of 3D bioprinted tissue constructs (Fig. 5c) [265]. Initially, thermal cross-linking was used to add hydrogen bonds to the SISMA-GO hydrogel, then subsequent photo cross-linking increased its stability by adding excess covalent bonds. These bonds protected the SISMA-GO hydrogel from excessive swelling and maintained a stable structure for a relatively extended period, making the SISMA-GO hydrogel suitable for 3D bioprinting (Fig. 5d) [273]. Human adipose-derived mesenchymal stem cells (hAD-MSCs) were then successfully bioprinted with the SISMA-GO hydrogel, demonstrating high cell viability over 7 d and highlighting the greater biocompatibility of the proposed material [273]. The *in-situ* reduction of GO (rGO) further enhanced the electrical conductivity of these nanostructures. The use of rGO is thus expected to improve cell differentiation and tissue maturation processes with the use of highly controllable electrostimulation strategies, while avoiding the current limitations of conventional electroconductive hydrogels, such as cytotoxicity and poor dispersibility [273].

Incorporating small intestine submucosa (SIS) into bone biomaterials has recently been attempted because SIS has proven to be effective for efficient bone repair by promoting angiogenesis and osteogenesis [266]. However, the excellent bioactivity of SIS is offset by its poor printability; in particular, SIS-derived bioink usually has low viscosity, meaning that the printed construct can quickly lose its original shape and collapse [273]. To address this issue and to better mimic the local bone environment, Yang et al. incorporated inorganic Sr^2+^/Fe^3+^ co-doped nHAps into the printing slurry of an SIS bioink [282], producing SIS/SrFeHA scaffolds with 3D interconnected macro/microporous patterns [274]. This platform exhibited a rough microsurface and improved mechanical strength, along with the synergic release of bioactive ECM components. These favorable physicochemical cues produced an SIS/SrFeHA composite with a highly biomimetic microenvironment for bone tissue. In addition, when the SIS/SrFeHA scaffold was coupled with human umbilical vein endothelial cells (HUVECs), the rich expression of RNA related to extracellular structural organization, ECM organization, proteinaceous ECM, angiogenesis, skeletal system development, and tube morphogenesis was observed (Fig. 5e) [282]. Based on the confirmed bone-healing potential of SIS/SrFeHA scaffolds, the incorporation of natural polymers with nanomaterials appears to represent a promising strategy for future bone TE applications.

The electrical conductivity of hydrogels can be raised by adding conductive nanofillers to the ECM hydrogel matrix [246]. The conductive materials bridge the insulating pore walls of the hydrogel, allowing for the propagation of electrical signals and stimulating cells within the 3D construct [275]. Sanjuan-Alberte et al. developed a conductive bioink by combining the electroconductive features of MWCNTs with human pluripotent stem cell-derived cardiomyocytes (hPSC-CMs) encapsulated in a bone-derived dECM hydrogel. Using this novel bioink, 3D bioprinting was conducted to fabricate cardiac tissue resembling a scaffold [267]. Following electrical stimulation (ES, a square wave, − 2 to 2 V, 1 Hz), the contractions of the hPSC-CMs on the printed scaffold were more defined and rhythmic [275]. Furthermore, in the presence of MWCNTs and ES, the contraction rate of hPSC-CMs was significantly higher compared with the MWCNT-dECM-derived control scaffold without ES [275]. These observations demonstrate the potential of this material to be used in the development of intelligent platforms for biosensing and actuating applications.

Hypertrophic scarring (HS) can occur during the pathological wound healing process and affect the appearance and physical activity of a patient [276]. Because existing treatment approaches to HS cannot meet the needs of patients, a skin substitute needs to be developed to promote wound healing and reduce the occurrence of HS. Therefore, Fang et al. aimed to create a bilayered membranous nanofiber scaffold (known as BLM) that was similar to the native skin structure [268]. In particular, an electrospun PLGA nanofibrous sheet was used to replicate the upper epidermis of native skin, while a 3D printed skin-derived dECM nanofiber scaffold was used as the lower dermis layer (Fig. 5f) [276]. It was found that the tensile stress, elongation at break, and Young's modulus of the dECM nanofiber hydrogel increased after the addition of the PGLA layer. Because the mechanical properties of the skin scaffold were critical to wound healing, the PLGA nanofiber layer provided efficient hydrogel protection and promoted skin tissue regeneration [276]. In addition, in vivo experiments showed that BLM nanofiber scaffolds inhibited collagen fiber deposition and angiogenesis by reducing CD31 positive signals, consequently inhibiting HS [276].

## Utilization of various nanobiomaterials for in vitro tissue-monitoring systems

A number of novel platforms that combine in vitro cell or tissue cultures with monitoring systems in a single layout have been proposed recently. Considerable effort has been devoted to developing cell-compatible in vitro tissue-integrated monitoring platforms using ECM hydrogels. Of the various ECM-based platforms that have been proposed, GelMA has emerged as the most popular choice of use, and it has been extensively combined with a range of nanomaterials, including CNTs [277, 281–283], rGO [284, 285], silver nanoparticles (AgNPs) [278], silica nanoparticles [286–288], and cellulose nanocrystals [289]. In addition to GelMA, other ECM hydrogels such as alginate [279, 280, 290] and ChiMA [280] have also been utilized for the fabrication of monitoring platforms, benefiting from their favorable mechanical properties (Table 7).

**Table 7:** 3D printed in vitro tissue-integrated monitoring platforms using ECM hydrogel-based nanobiomaterials.

Base ECM	Nanocomponent	Function of the nanocomponent	Application (Type of printing)	Refs.
GelMA	Self-assembled flower-like copper oxide nanoparticles (FCONp) and hydrazide-functionalized multiwalled carbon nanotubes (MWCNT-CDH)	Electrical conductivity enhancer	3D intestinal microvillus-mimetic electrochemical cell sensor for detection of allergen (stereolithography-based 3D printing)	[277]
	Silver nanoparticles (AgNPs)	Electrical conductivity enhancer	Observation of (malignant) cell and tissue (extrusion and drop-on-demand microvalve-based printing)	[278]
Alginate/methyl-cellulose	O_2_-sensitive luminescent styrene-maleic anhydride copolymer (PSMA) nanoparticles	Luminescent indicators for O_2_ concentration	Non-invasive, optical observation of oxygen dynamics and metabolic activity of cell and tissue (extrusion-based 3D printing)	[279]
Methacrylated chitosan (ChiMA)	Chemically converted graphene (CCG) nanosheets	Mechanical reinforcer Electrical conductivity enhancer	Biodegradable conducting substrates (Extrusion-based 3D printing)	[280]

**Fig. 6:**
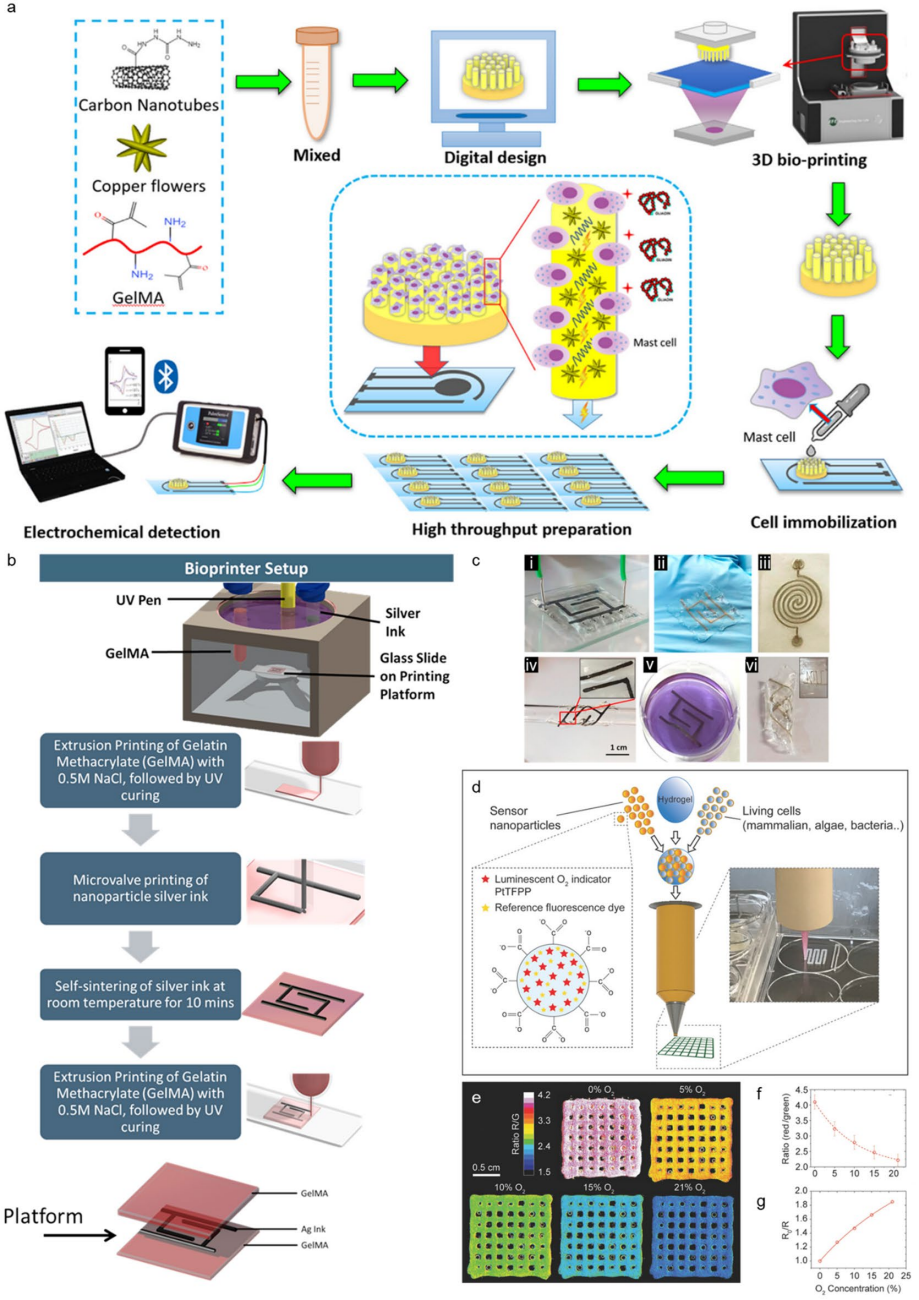
Fabrication of various ECM hydrogel-based nanobiomaterial ink for in vitro tissue-integrated monitoring platforms using different 3D printing techniques.** a** Schematic diagram of the fabrication process of the 3D intestinal microvillus-mimetic electrochemical cell sensor. Reprinted with permission from [277]. **b**, **c** Diagram illustrating the bioprinter setup for the one-step fabrication of a bioelectronics platform and the resulting freestanding and flexible sensor: **b** comprehensive 3D bioprinting process and **c** images of the bioelectronic platforms printed (i) on glass, (ii) in freestanding form, and (iii) the heating coil; (iv) the printed electrical circuits are flexible enough to be conformally wrapped around a cylinder into a conformal shape and (v) stable even when immersed in a wet environment, while (vi) the heating coils printed on GelMA are also flexible. Reprinted with permission from [278]. **d–g** Novel approach for the fabrication of an oxygen-sensitive sensor using 3D printing and functionalized ink:** d** schematic diagram illustrating the printing process for the oxygen sensor using O_2_-sensitive luminescent indicator-incorporated ink, **e** images of a 3D-printed optical oxygen sensor at various oxygen concentrations,** f** calibration curve obtained from **e**, and **g** a Stern–Volmer (fluorescence quenching) plot illustrating the calibration curve. Reprinted with permission from [279]

Jiang et al. developed a composite hydrogel based on GelMA, MWCNTs, and copper oxide nanoparticles to mimic the microvillus structures of the small intestine to mimic the food-derived allergic reactions (Fig. 6a) [277]. The CNTs were modified with positive charges using hydrazide functionalization to achieve a stable dispersion within the GelMA hydrogel, while copper oxide nanoparticles were incorporated to enhance electrical signal amplification. The resulting GelMA-based composite hydrogel was utilized to fabricate the villus structure, and mast cells were subsequently immobilized onto the villus. Because the mast cells have receptors for allergens, a series of intracellular activities was triggered altering the biochemical properties of the cells when allergens were recognized, which in turn influenced the electrical signals. By recapitulating the 3D structure of the human small intestinal villus, the engineered tissue and the monitoring platform exhibited enhanced cellular functionality compared to traditional 2D cell sheet systems.

In a study conducted by Agarwala et al., a freestanding and flexible bioelectronic platform was fabricated using a GelMA-based composite ink blended with AgNPs (Fig. 6b,c) [278]. The platform consisting of microelectrodes and heating coils was printed using AgNPs and GelMA nanobiomaterial ink and embedded within two GelMA layers. This design allowed for electrical stimulation and the controlled heating of the cells while providing insulation to ensure stability in a wet environment. Unlike previous studies, the researchers simultaneously employed both extrusion printing and drop-on-demand microvalve-based printing techniques to fabricate a biocompatible hydrogel component alongside the electronics component. This approach enhanced the efficiency of multi-material printing, allowing for the integration of a diverse range of materials in a single platform.

Trampe et al. developed a non-invasive strategy for identifying and quantifying hypoxia within 3D-printed tissue constructs of clinically relevant dimensions using O_2_-sensitive nanoparticles (Fig. 6d-g) [279]. Styrene-maleic anhydride copolymer (PSMA) nanoparticles were blended into alginate as luminescent indicators for O_2_ concentration and printed into mesh structures. The sensor-functionalized alginate ink enabled the optical mapping of the O_2_ distribution. Sayyar et al. developed chemically converted graphene (CCG) nanosheets and a ChiMA composite and used this nanobiomaterial ink to print multilayered lattice films [280]. Although the specific application of this study was not explicitly mentioned, the implementation of CCG resulted in the successful development of a biodegradable conducting substrate film to which cells can adhere and grow. In addition, this film exhibited superior mechanical strength compared to pure ChiMA.

Even though 3D printing enables the fabrication of intricate tissue constructs and monitoring platforms by using multiple materials [291–293], the application of 3D printing technology as a fabrication method in this field remains relatively limited. This can be attributed to the tendency of nanoparticles to self-aggregate, likely due to van der Waals interactions. Furthermore, in the fabrication of monitoring systems, a significantly higher volume of nanomaterials is required compared to tissue fabrication, exacerbating aggregation and nozzle-clogging issues. Additionally, the inhomogeneous dispersion of nanoparticles presents a formidable challenge, including the undesirable concentration of stress and impeded load transfer within the composite system. To mitigate this issue, enhancing nanomaterial-polymer network interaction, such as through dopamine/catechol functionalization, may promote a more uniform dispersion. [205, 283, 294].

## Future perspectives

Despite the promising potential of nanocomposites in TE and RM, several challenges must be addressed before their widespread use. First, safety concerns must be carefully evaluated to confirm the biocompatibility of proposed nanobiomaterials and nanobioinks. For example, the cellular processes involved in identifying CNTs and how the physical and chemical characteristics of CNTs influence their toxicity are not yet completely understood, and further research is required [295, 296]. It is particularly important to be aware of issues related to the use of nanomaterials because many of them are not biodegradable and can pose significant risks to human health [296–300]. Additional studies are needed to establish the cellular mechanisms and potential long-term effects of these systems on host tissues. Therefore, when considering nanomaterials in TE, it is crucial to consider the effect of their size, synthesis method, shape, purity, solubility, concentration, and aggregation, which can govern their mechanical and biological performance [301–304]. In addition, efforts must be focused on developing and implementing standardized safety assessments to facilitate the transition of nanocomposite-based TE approaches into clinical practice.

Second, 3D bioprinting technology has distinct advantages in the fabrication of complex structures, such as a multilayer tubular structure in a single step [75, 156, 305]. However, mixing nanoparticles into printing ink may present challenges, for instance, nozzle clogging during printing [306–311], which explains why this technology has not been widely adopted in the nano field. Nevertheless, several studies have successfully printed nanocomposite ink by finely adjusting various printing parameters [308, 312, 313]. Thus, optimizing the printing conditions for nanobioinks is a promising strategy that enhances the functional benefits of nanocomposites [314–316].

Finally, while advanced ECM materials and fabrication techniques have been employed to mimic tissue-specific properties, this has been lacking for nanomaterials. For example, electroactive tissues such as cardiac muscle, skeletal muscle, and nerves communicate through intercellular electrical signaling. Although researchers have explored the incorporation of various nanomaterials into ECM-based hydrogels to enhance their electrical conductivity, there has been a lack of tissue-specific design. Therefore, it is necessary to consider the tissue-specific design of nanomaterials to enhance the functionality of in vitro tissues within a hybrid system.

## Data Availability

Not applicable.

## Abbreviations

TE: Tissue engineering
RM: Regenerative medicine
ECM: Extracellular matrix
3D: Three-dimensional
dECM: Decellularized extracellular matrix
ColMA or MeCol: Collagen methacryloyl
FDA: Food and Drug Administration
RGD: Arginine-glycine-aspartic acid
GelMA: Gelatin methacryloyl
ChiMA or MAC: Chitosan methacryloyl
HA: Hyaluronic acid
SF: Silk fibroin
GNW: Gold nanowire
CNT: Carbon nanotube
nHAp: Nanohydroxyapatite
GNR: Gold nanorod
DAC: Dialdehyde cellulose
CNF: Cellulose nanofibrils
AuNP: Gold nanoparticle
CNC: Cellulose nanocrystal
BSA: Bovine serum albumin
VEGF: Vascular endothelial growth factor
NSC: Neural stem cell
GO: Graphene oxide
PLA: Polylactic acid
iPSC: Induced pluripotent stem cell
MWNT: Multiwalled carbon nanotube
hASC: Stem cells derived from human adipose tissue
CMC: Carboxymethylcellulose
HEK293: Human embryonic kidney 293 cell
LNP: Lignin nanoparticles
M: Million
SCI: Spinal cord injury
WHO: World Health Organization
HAMA: Hyaluronic acid methacryloyl
PEDOT: Poly(3,4-ethylene-dioxythiophene)
LS: Sulfonated lignin
Sr2 +: Strontium
Tuj-1: Neuron-specific class III beta-tubulin III
GFAP: Glial Fibrillary Acidic Protein
CNP: Cellulose nanoparticle
dECM-MA: Methacrylate reaction
PLGA: Poly(lactide-co-glycolide);MHC: Myosin heavy chain
SISMA: Methacryloyl-modified, small intestine submucosa-derived dECM
rGO: GO reduction
HUVEC: Human umbilical vein endothelial cells
hPSC-CMs: Human pluripotent stem cell-derived cardiomyocytes
ES: Electrical stimulation
HS: Hypertrophic scar
BLM: Bilayered membranous
KEGG: Kyoto Encyclopedia of Genes and Genomes
FCONp: Flower-like copper oxide nanoparticle
MWCNT-CDH: Hydrazide-functionalized multiwalled carbon nanotube
AgNP: Silver nanoparticle
PSMA: Styrene-maleic anhydride copolymer
CCG: Chemically converted graphene
